# *Drosophila* oocyte proteome composition covaries with female mating status

**DOI:** 10.1038/s41598-021-82801-4

**Published:** 2021-02-04

**Authors:** Caitlin E. McDonough-Goldstein, Scott Pitnick, Steve Dorus

**Affiliations:** Center for Reproductive Evolution, Biology Department, Syracuse University, Syracuse, NY USA

**Keywords:** Proteomic analysis, Oogenesis

## Abstract

Oocyte composition can directly influence offspring fitness, particularly in oviparous species such as most insects, where it is the primary form of parental investment. Oocyte production is also energetically costly, dependent on female condition and responsive to external cues. Here, we investigated whether mating influences mature oocyte composition in *Drosophila melanogaster* using a quantitative proteomic approach. Our analyses robustly identified 4,485 oocyte proteins and revealed that stage-14 oocytes from mated females differed significantly in protein composition relative to oocytes from unmated females. Proteins forming a highly interconnected network enriched for translational machinery and transmembrane proteins were increased in oocytes from mated females, including calcium binding and transport proteins. This mating-induced modulation of oocyte maturation was also significantly associated with proteome changes that are known to be triggered by egg activation. We propose that these compositional changes are likely to have fitness consequences and adaptive implications given the importance of oocyte protein composition, rather than active gene expression, to the maternal-to-zygotic transition and early embryogenesis.

## Introduction

For species with minimal parental care, such as the majority of insects and other invertebrates, parental investment predominantly consists of the material invested in oocytes^1,2^. Variation in oocyte investment therefore has a direct and substantial impact on early development and hence viability. Oocyte quantity and quality have also been shown to vary in response to female condition^3–5^. Thus, allocation mechanisms may evolve to maximize female expenditure into oocytes when fertilization opportunities are abundant and environmental conditions are favorable for offspring survival.

Direct transfer of nutrients to the female during mating is one adaptive strategy by which males can influence oocyte attributes. In a variety of insects, males provide nuptial gifts of nutritional resources through ejaculate components, secretions or body parts^1,6,7^. Intraspecific variation in nuptial gift quality and quantity has experimentally been shown to correspond with numbers of oocytes^8–13^ and oocyte size^9,12,14^, as well as offspring maturation time^14^, size, and lifespan^15^. Supporting female utilization of male-derived nutrients in oogenesis are observations that ejaculate components become incorporated into the oocytes or ovaries of multiple fruit fly species^16,17^, butterflies and moths^18,19^, stink bugs^20^, cockroaches^21^, weevils^22^, lampyrid beetles^23^, and grasshoppers^24^. However, we note that in *D. melanogaster* male-derived chemical elements, but not ejaculate proteins, have been detected in oocytes^17,25^. Another strategy is male stimulation of increased female investment into oocytes following mating. Seminal fluid proteins of diverse insect species have been found to influence oocyte production through the stimulation of endogenous, female-mediated oogenesis mechanisms^26,27^. For example, ejaculate receipt by females can stimulate expression of vitellogenins necessary for oocyte production^28^, increased oocyte size^29,30^ and increased quantity of oocytes oviposited^31^.

Here, we investigated whether female mating status influenced the protein composition of *D. melanogaster* oocytes. We compared the proteome of stage 14 oocytes between those matured in unmated versus mated females. As these oocytes were not ovulated or fertilized, protein differences detected were primarily attributable to the influence of mating on oocyte development. This proteomic variation indicates that mating is likely to alter female-mediated aspects of oocyte maturation and provides insights into maternal investment strategies.

## Results

### Characterization of the mature oocyte proteome with respect to female mating status

**Figure 1:**
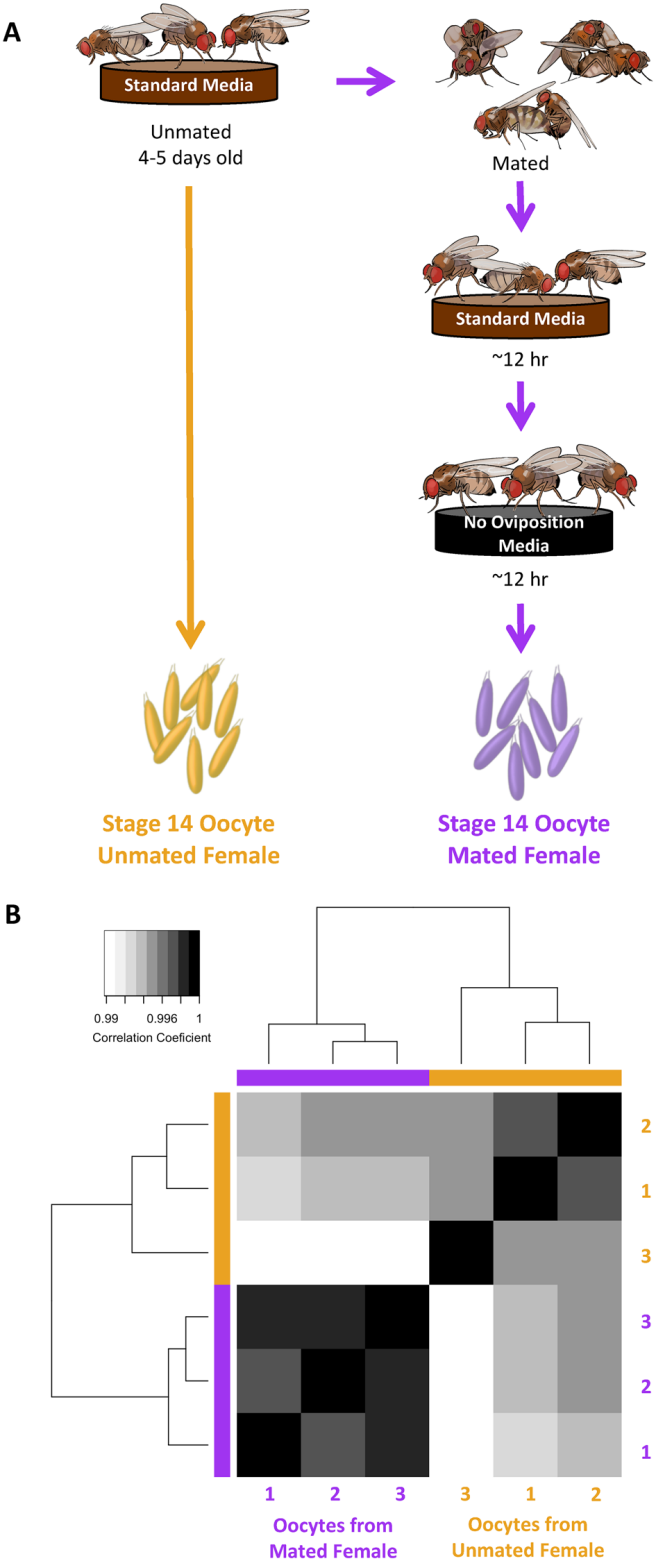
Experimental design and hierarchical clustering of oocyte proteomes. (**A**) Schematic of experimental design (image permission: Siyuan Cong). Oocytes were collected either from unmated females (left) or mated females (right). After mating, females were allowed to ovulate and clear out oocytes that had matured prior to mating and then transferred to a standard no oviposition media for 12hrs to accumulate oocytes that matured in a mated female environment. (**B**) Euclidean distance hierarchical clustering of oocytes proteome replicates based on protein abundance.

We characterized the proteome of mature oocytes (stage 14) collected from mated and unmated females (Fig. 1A). Overall, we identified 4,485 high confidence oocyte proteins, with proteome composition highly reproducible across replicates (all pairwise protein abundance correlations were greater than 95%; Fig. 1B). These results were consistent with previous studies in terms of proteome coverage, including an 89% overlap with previously described *D. melanogaster* oocyte proteomes^32–34^. The three most abundant proteins were yolk proteins (Yp1, Yp2, Yp3*)*, which have previously been shown to be the predominant protein component of oocytes^35^. Heat shock proteins (Hsp83 and Hsp26), which have also been shown to accumulate in the oocyte during oogenesis and contribute to oocyte stability^36^, were also among the top 20 most abundant proteins. The top 10% of oocyte proteins by abundance had Gene Ontology (GO) enrichments for: microtubule associated complex (GO:0005875, *p* < 0.001), lipid particle (GO:0005811, *p* < 0.001), proteasome complex (GO:0000502, *p* = 0.004), translation (GO:0006412, *p* < 0.001), protein folding (GO:0006457, *p* < 0.001), and centrosome duplication (GO:0051298, *p* < 0.001). Notable amongst these abundant proteins were those critical to oocyte differentiation or embryo viability (i.e., tudor, vasa, abnormal spindle, and belle)^37–40^.

**Figure 2:**
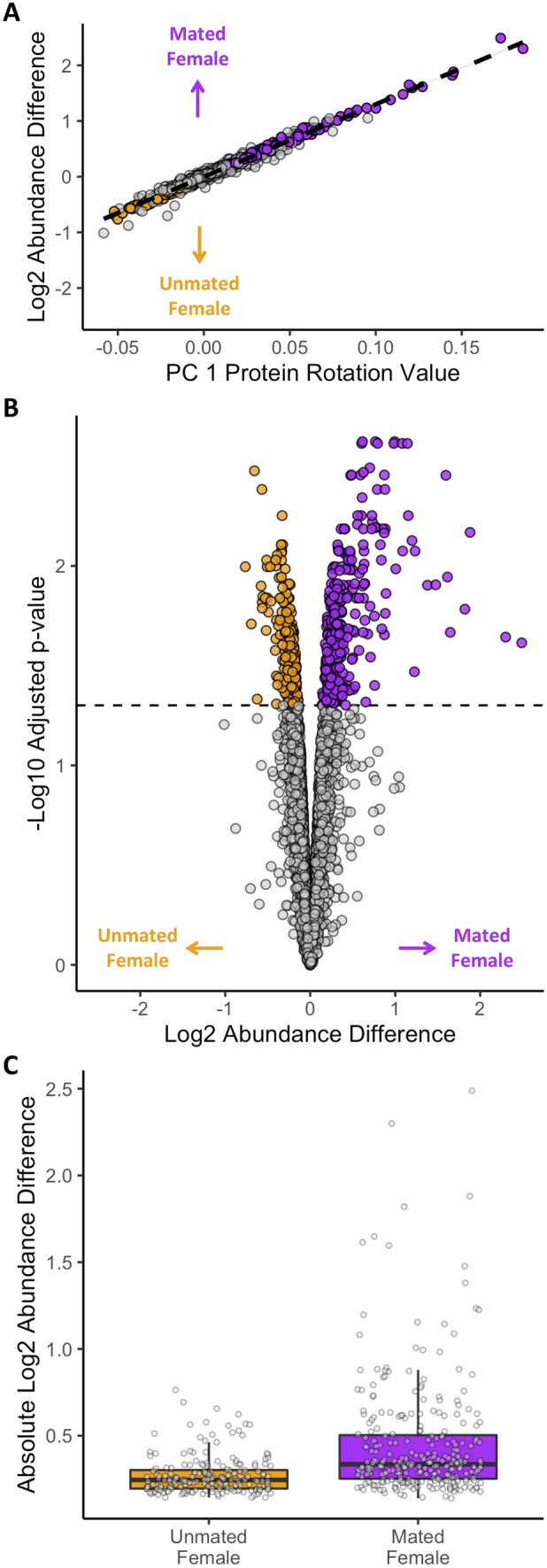
Oocyte proteome composition differences were dependent on female mating status. (**A**) Linear correlation between principle component 1(PC1) protein loadings with differential abundance between oocytes from unmated and mated females (log2 fold abundance difference) indicates that this axis of variation is associated with mating status. Colored points indicate proteins with significant differential abundance. (**B**) Volcano plot of log2 fold differential protein abundance between oocytes from unmated and mated females and -log10 adjusted *p*-values. (**C**) Boxplot of log2 fold abundance differences of proteins exhibiting significant abundance differences in oocytes from either unmated or mated females. A significantly greater magnitude of abundance change (*p* < 0.001) was observed in proteins with greater abundance in oocytes from mated females.

To test whether oocyte proteome composition was influenced by female mating status, we compared protein abundance in mature oocytes from unmated females to those from females 24 h after mating. Principal components (PC) analysis revealed that the first PC (59.2% of variation explained) distinguished oocyte samples by mating status (Fig S1). PC1 rotation values were significantly correlated with the log fold difference in abundance between oocytes from mated and unmated females (R^2^ = 0.98, *p* < 0.001), confirming that PC1 captured variance associated with mating status (Fig. 2A). We next identified 496 proteins that were significantly differentially abundant between oocytes from unmated and mated females (Fig. 2B). Altogether, 11.1% of the proteome exhibited significant abundance differences, including 20 proteins that exceed a two-fold change in abundance. These differentially abundant proteins were significantly biased towards greater abundances in oocytes from mated females (59.7%; binomial probability, *p* < 0.001) and there was a larger magnitude of protein abundance increases in mated female oocytes relative to unmated (mean log2FC of 0.45 ± 0.02) versus unmated (mean log2FC of 0.27 ± 0.01); Kruskal-Wallace χ^2^ = 357.34, *p* < 0.001; Fig. 2C). These differences in protein composition indicate that oocyte content is influenced by female mating status.

### Oocyte proteome variation is not consistent with follicle cell contributions or aging

**Figure 3:**
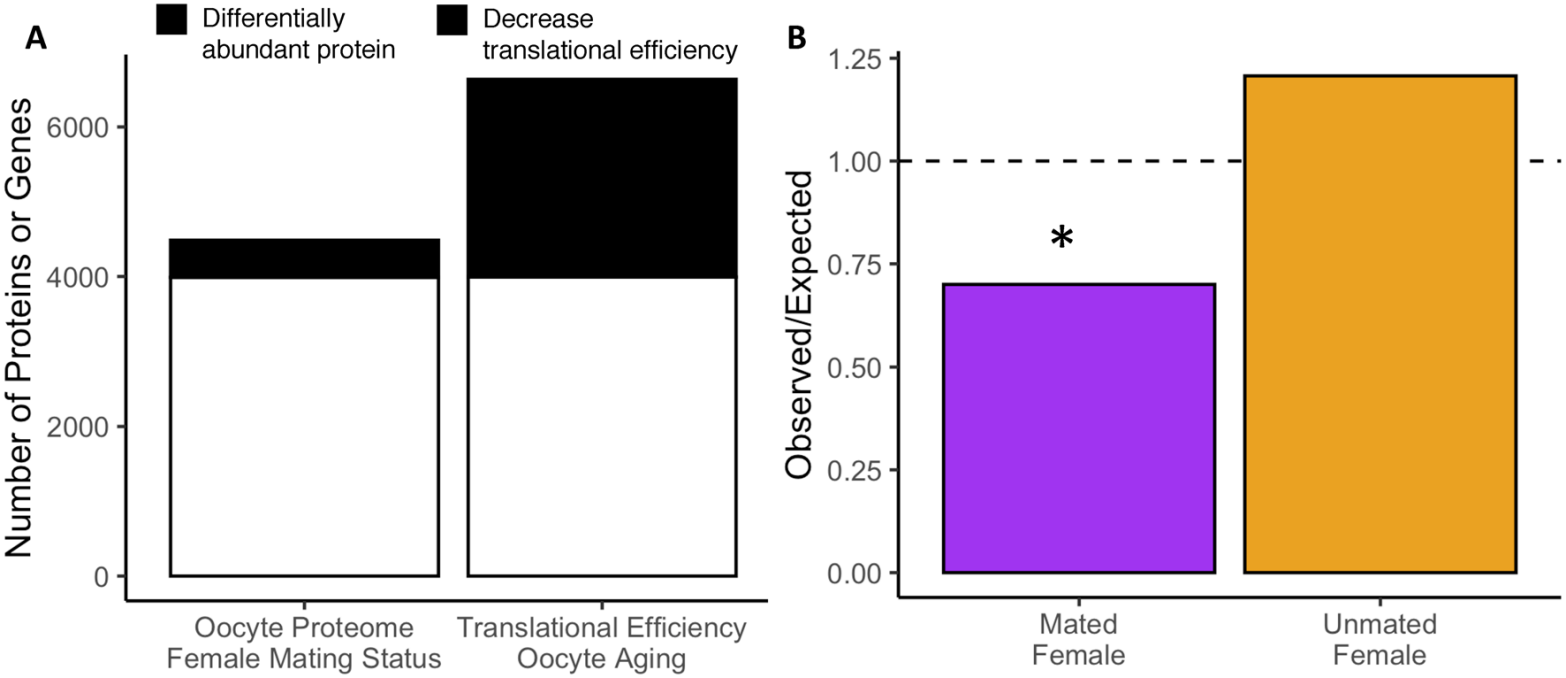
Oocyte proteome composition differences are distinct from age-dependent changes. (**A**) Stacked bar chart displaying the proportions of oocyte proteins exhibiting mating-dependent changes in abundance and genes exhibiting age-dependent decreases in translational efficiency. (**B**) Observed versus expected overlap of proteins exhibiting age-dependent reductions in translational efficiency and proteins that change in abundance between oocytes from mated and unmated females (*p < 0.001).

To ensure that the identified oocyte proteomic differences were due to female mating status, we evaluated two alternative hypothesized mechanisms: (1) that proteome differences were due to changes in follicle cells surrounding the oocyte rather than the oocyte itself and (2) that age-dependent oocyte effects contributed to the observed differences (i.e., the duration an oocyte remains in the ovary before ovulation). To address the first alternative hypothesis, we examined proteins commonly identified in follicle cells^33,34^ and found that they comprise less than 1% of the oocyte proteome (40 out of 4485 proteins) and were not significantly enriched among differentially abundant proteins (lower-tail binomial cumulative probability test *p* = 0.63; Table S1). To address the second alternative hypothesis, we assessed whether proteomic differences in oocytes from unmated and mated females corresponded to age-dependent changes in translational efficiency that would be expected to influence proteome composition^41^. Our experimental design necessitated consideration of this possibility because oocytes from unmated females could range from 0 to 96 h old, whereas those from mated females were 24 h old or less. However, we note that oocyte age differences in Greenblatt et al. were not accompanied by changes in oocyte protein content or hating rate. A comparison of our proteomic observations to the global patterns of reduced translational efficiency in aging oocytes revealed three primary differences^41^. First, in contrast to age-dependent reductions in translational efficiency which were widespread (39.8%; 2644 out of 6637), mating-dependent changes in the oocyte proteome were significantly more specific in nature (11.1%; 496 out of 4,485) (Fig. 3A; Table S1). Second, while age-dependent changes overwhelmingly resulted in reduced translational efficiency (97.7%, 2644 out of 2707), mating-dependent changes were significantly more balanced in their direction of change (59.7% or 296 out of 496 exhibited increased abundance in oocytes of mated females; χ^2^ = 1065.2, *p* < 0.001). Third, proteins with lower abundance in potentially older oocytes from unmated females (i.e., those with greater abundance in potentially younger oocytes from mated females) were significantly underrepresented among genes with age-dependent decreases in translational efficiency (lower-tail binomial cumulative probability test *p* = 0.03, expected 134.0, observed 94 proteins differentially abundant proteins with decreased translational efficiency; Fig. 3B). These results indicated that our proteomic observations were distinct in magnitude, direction and composition from translational efficiency reductions associated with aging. Instead, we conclude that the observed proteomic variation is more likely to be primarily due to differences in oocyte maturation between mated and unmated females. However, we also note that mated females had to be kept on no oviposition media for 12 h and that this nutritional variable may also impact oogenesis.

### Highly connected protein networks contribute to oocyte proteome variation

**Figure 4:**
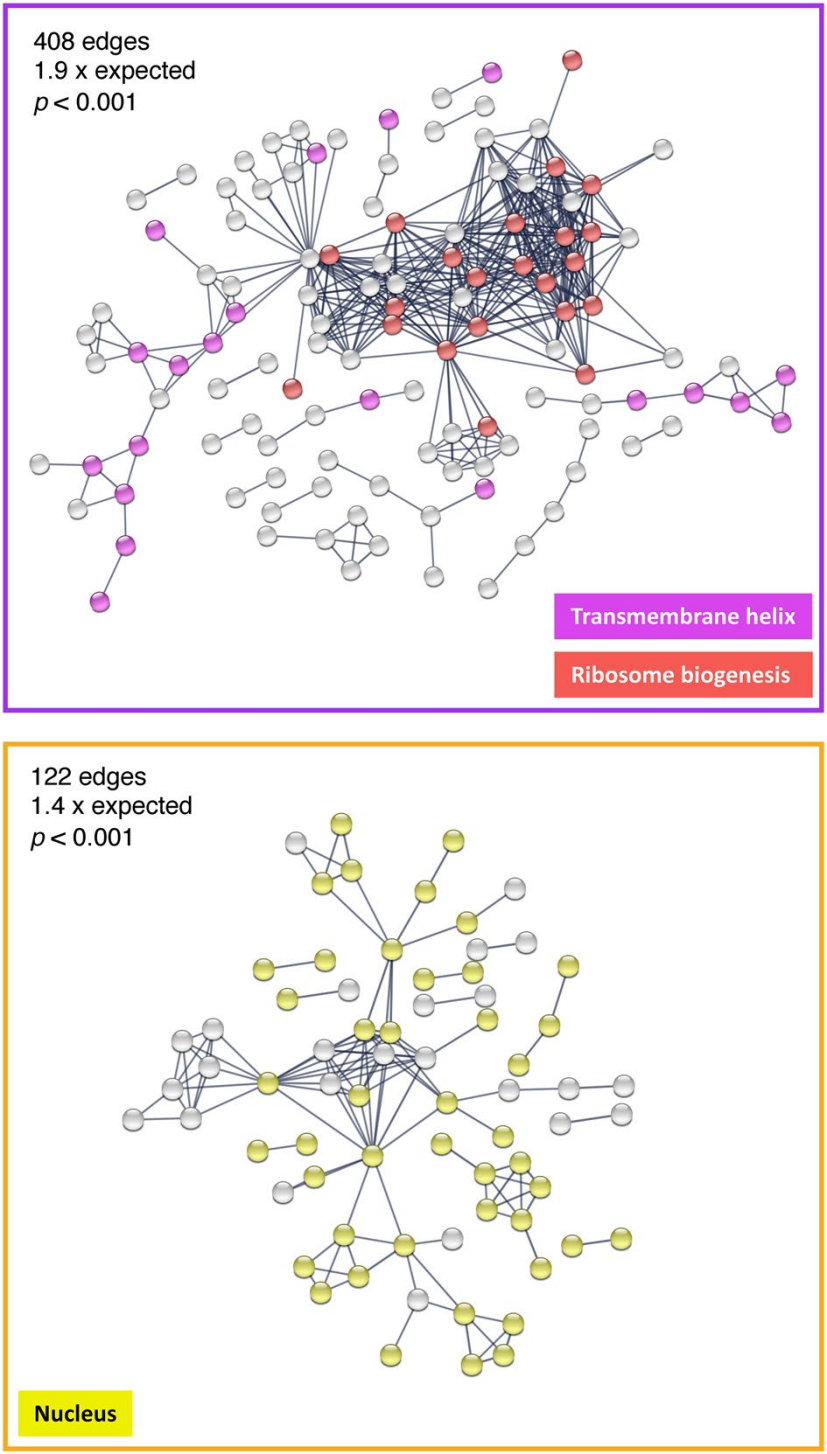
Network enrichment of differentially abundant oocyte proteins. Differentially abundant proteins were analyzed for connectivity and functional enrichment using high confidence protein interactions (confidence > 0.9; line thickness indicates strength of support for protein interactions). Proteins with increased abundance in oocytes from mated (top) and unmated (bottom) females had significantly more interactions than expected. Proteins with greater abundance in oocytes from mated females were significantly enriched for ribosome biogenesis (red) and transmembrane proteins (purple) whereas proteins with greater abundance in oocytes from unmated females were significantly enriched for associations with the nucleus (yellow).

We investigated the functional coherence of mating-dependent changes in oocyte proteome composition using GO enrichment analyses (Table S2). Proteins more abundant in oocytes from mated females exhibited enrichments for integral membrane component (GO: 0016021, *adj. p* < 0.001), nucleolus (GO:0005730, *adj. p* < 0.001) and endomembrane system (GO:0012505, *adj. p* < 0.001). Proteins more abundant in oocytes from unmated females exhibited enrichments for components of the nucleus (GO:0005634, *adj. p* = 0.006). We next investigated patterns of functional enrichment among differentially abundant oocyte proteins using protein network interaction information. Proteins with greater abundance in oocytes from mated females (296 proteins) comprised a significantly interconnected protein network (PPI enrichment *p* < 0.001) with 1.9 times more interactions per node than expected (408 edges observed and 210 edges expected; Fig. 4) This protein network was enriched for ribosome biogenesis (GO: 0042254, *adj. p* = 0.03) and proteins with a transmembrane helix (KW-1133, *adj. p* < 0.001; Table S2). The potential importance of ribosomal protein accumulation is supported by studies showing high rates of ribosome synthesis in the ovary, the reduced size of oocytes when ribosomal proteins are mutated, and longer oocyte development times when rRNA levels are disrupted^42,43^. It is also plausible that elevated amounts of translational machinery could result in oocytes better primed for egg activation, which would influence embryonic development prior to zygotic genome activation^44^. The enrichment of proteins containing transmembrane helixes included membrane proteins involved in calcium-related processes. Calcium influx is the critical trigger for egg activation^45^ and several of these membrane proteins were involved in calcium channel activity (i.e., *painless*, *Tmem63*), calcium ion binding (i.e., alpha-Man-la, AnxB11, CG17271, CG17272, Edem2, LPCAT, mgl, Ndg, qua) and calcium homeostasis (i.e., *CG6230*).

For proteins with greater abundance in oocytes from unmated females (200 proteins), we also observed protein networks with significant interconnectivity, including 1.4 times the expected interactions among proteins (PPI enrichment *adj. p* < 0.001; 122 edges observed 85 expected). However, the average protein connectedness was less than half that of proteins found to be more abundant in oocytes from mated females (1.22 average for proteins greater in oocytes from unmated females vs. 2.77 average for proteins greater in mated females; Fig. 4). The network of proteins with greater abundance in oocytes from unmated females was significantly enriched for association with the nucleus (GO: 0005634, *adj. p* < 0.001) and chromosomes (GO:0098687, *adj. p* = 0.005). This network further included proteins involved in chromosome segregation, such as microtubules, spindle, kinetochore, and centriole, that could contribute to the resumption and completion of meiosis or mitosis following egg activation^32,33^.

### Relationship between oocyte maturation and mating-induced changes in oocyte composition

The observed proteomic variation was likely to have occurred during oocyte maturation, the last stage of oogenesis (release from prophase I arrest to metaphase I arrest) when substantial changes to oocyte composition occur^46^. Previous characterization of maturation-associated proteome changes (i.e. between stage 11 vs. stage 14 of oocyte development) found that approximately 30% of the proteome changed in abundance^33^. Proteins that increased during maturation were enriched for functions related to meiotic progression, whereas proteins that decreased were enriched for translational machinery that may degrade concomitantly with nurse cells. To evaluate whether female mating status influenced oocyte maturation, we next investigated how our results correspond to the established global proteomic changes associated with maturation (Table S1).

**Figure 5:**
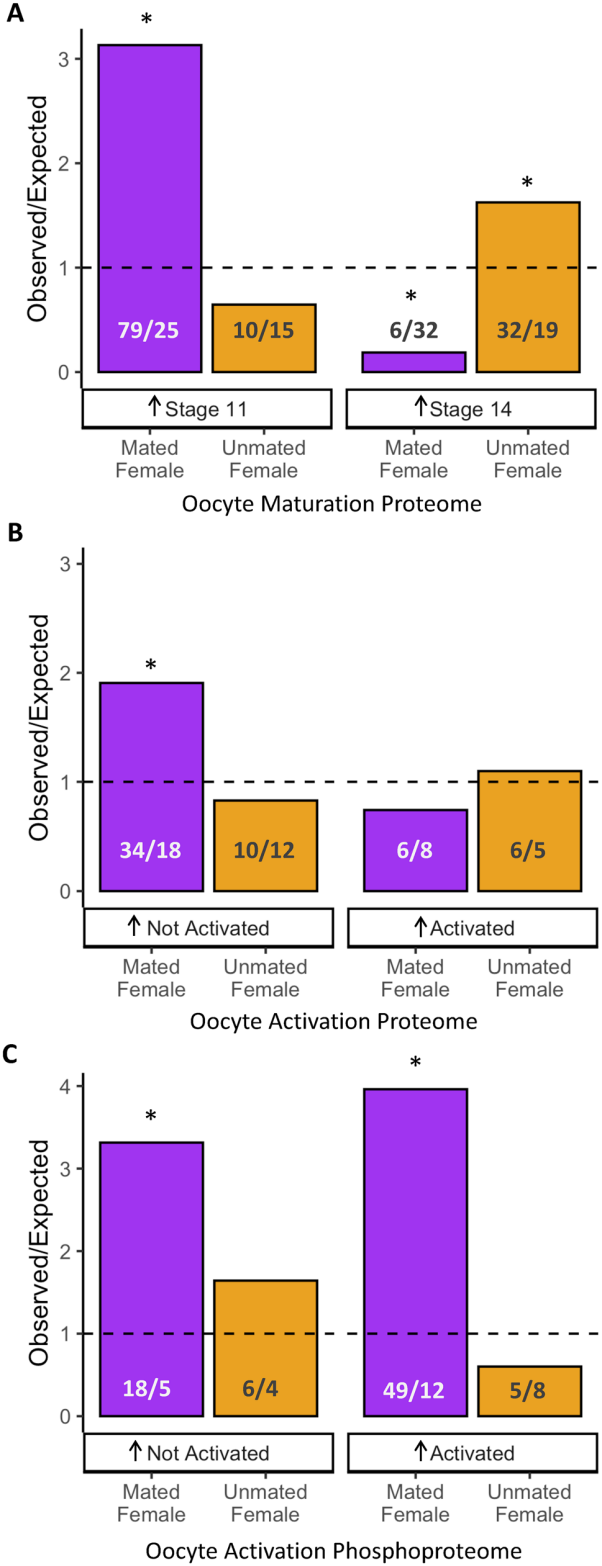
Comparison to protein changes that occur during oocyte maturation and activation. Bar plots of the observed versus expected protein overlap between protein abundances differences in oocytes from mated (purple) and unmated females (orange) with (**A**) proteomic changes during oocyte maturation, (**B**) proteomic changes during oocyte activation and (**C**) proteomic changes in phosphoproteins during oocyte activation. The number of observed versus expected (rounded to the nearest whole number) proteins is indicated. Significance is indicated by asterisks (*p < 0.01).

We identified a fairly weak, albeit significant, negative correlation between protein abundance differences during maturation and protein abundance differences associated with mating status (r = − 0.25, df = 4131, *p* < 0.0001; Fig S2). Next, we focused specifically on the overlap between proteins exhibiting mating-dependent abundance differences to those observed to change in abundance during oocyte maturation (Fig. 5A). Among proteins that increase in abundance during maturation (i.e., greater abundance in stage 14 oocytes), we observed a larger than expected overlap with proteins that had greater abundance in oocytes from unmated females (upper-tail cumulative probability test *p* = 0.002). In contrast, proteins that decrease in abundance during maturation (i.e., greater abundance in stage 11 oocytes) exhibited a significantly larger than expected overlap with proteins that had greater abundance in oocytes from mated females (upper-tail cumulative probability test *p* < 0.001). Notably, 25% of the overlapping proteins that decreased in abundance during maturation and were more abundant in oocytes from mated females had a transmembrane domain. Thus, mating status does appear to influence oocyte maturation dynamics, particularly in relation to transmembrane proteins.

### Relationship between egg activation and mating-induced changes in oocyte composition

Following maturation, the next transition for oocytes is activation, which involves the resumption of meiosis as the ovulated oocyte passes through the oviduct^47^. Egg activation is a transcriptionally silent process and characterized by dynamic changes in protein abundance and phosphorylation state^32,34^. Kronja et al. found that many proteins that increased in abundance during egg activation were important for embryonic development, such as proteins involved in chromosome organization and replication. We compared our data to proteomic changes during activation to evaluate the extent to which oocyte composition differences associated with mating status could influence post-activation dynamics (Table S1). We observed an enrichment for proteins that decreased in abundance during activation and had greater abundance in oocytes from mated females (upper-tail cumulative binomial probability test *p* < 0.001; Fig. 5B). Notably, the majority of these proteins (61.8%; 21 out of 34) also decreased in abundance during maturation (see above)^33^. Thus, proteins that normally decrease in abundance during maturation and activation appear to do so to a lesser extent in already mated females.

Finally, we compared our data to an analysis of changes in the phosphoproteome (i.e., proteins that are phosphorylated) during egg activation (Table S1)^34^. We found that phosphorylated proteins that changed in abundance (both increased and decreased) were more likely than expected to be of greater abundance in oocytes from mated females (increased at activation upper-tail cumulative binomial probability test *p* < 0.001; decreased at activation upper-tail cumulative binomial probability test *p* < 0.001; Fig. 5c). Thus, mating-associated changes in oocyte composition could influence downstream proteome dynamics following egg activation.

## Discussion

Mating induces a myriad of postmating physiological responses in *Drosophila* females, including the stimulation of oocyte maturation and increased rates of oogenesis^28,31,48^. Our analyses suggest that, concomitant with these responses, there are functionally coherent changes in oocyte proteome composition. Although the mechanisms responsible for these differences remain to be elucidated, the stimulation of oocyte maturation may accelerate the temporal progression of oogenesis and this, in turn, may alter the dynamics of protein translation and degradation, as well as the duration of interactions with nurse and follicle cells^49^. Our analysis suggests that mating-induced changes influence proteome dynamics associated with maturation, including transmembrane proteins. Intriguingly, we also found that mating-induced changes also bear a significant relationship to molecular hallmarks of egg activation^32,33^ and may therefore have downstream effects on fertilization success and early embryogenesis. Syncytial embryonic development in *Drosophila* is a highly coordinated process that relies heavily on maternal contributions^50^. Although zygotic gene expression occurs far earlier than previously recognized^51^, many aspects of pre-cellular development are likely to be programmed during oogenesis and thus may be influenced by variation in oocyte protein composition. At this time we cannot predict the potential functional ramifications of this variation but we note that protein abundance variation was relatively widespread (11.1% of proteins), although only a relatively small set (20 proteins) exceeded two-fold changes in abundance.

Two hypotheses address the possible adaptive value of mating-dependent modulation of oocyte composition. First, the acceleration of oogenesis in response to mating may be energetically costly. Investment into oocytes may therefore trade off with the quantitative rate of oocyte production. Under this scenario, it may be adaptive for females to maximize their investment in oocyte number and only transition to investing in the final stages of oogenesis after mating when oocytes can be fertilized. Note that this hypothesis assumes a fixed total energy available for oogenesis. An alternative hypothesis is that mating triggers a net increase of female investment in oogenesis, thus increasing both oocyte quantity and quality. Testing these "adaptive maternal investment" hypotheses will prove challenging. Nevertheless, fitness consequences of the mating-induced changes could be examined by comparing differences in offspring development and fitness indexes (e.g., lifetime fecundity of daughters and competitive mating/fertilization success of sons) between offspring from oocytes matured in an unmated female (i.e., the first cohort of eggs laid immediately after mating) with eggs laid at later timepoints when mating-induced aspects of oogenesis are manifest. We also predict that variation in oocyte investment will be dependent on interactions with male genotype or phenotype. Female × male interactions have been shown to extensively influence postmating reproductive events in *D. melanogaster*^52^, including egg volume^53^. In addition, male quality may also influence oocyte investment. For example, the stimulation of egg laying by older males is reduced relative to younger males, which may correspond with age-related changes in ejaculate composition^54,55^.

The phenomenon we demonstrate here is also likely to differ among species, with variation arising through selection associated with oocyte production/oviposition strategies and mating system evolution. For example, there is dramatic variation in the relative and absolute size of oocytes and in the pattern of oviposition (i.e., clutching versus continuous production and oviposition) among *Drosophila* species^56,57^ and insects in general^58^. Further, female remating behavior can vary dramatically, ranging from multiple matings each day, to a remating latency of multiple days, to a single mating per lifetime^59^. Species that remate rarely might exhibit more pronounced modulation of oocyte investment to optimize reproductive potential when they have had the opportunity to mate. Alternatively, those with greater competition may have greater differential postmating responses based on male quality. A comparative evolutionary approach may be valuable to understand the potential for adaptive maternal investment in species with frequent remating where mechanisms of cryptic female choice are prominent or selection favors a balance in investment across current and future mating opportunities.

## Methods

### Fly maintenance and sample preparation

Wildtype *D. melanogaster* LH_M_ strain was maintained in standard laboratory conditions at room temperature (~ 23 °C) with a natural light cycle. Flies were reared in glass bottles on a yeast, cornmeal, agar, and molasses media. Unmated females were collected and matured in vials of 10–15 flies with 1.5 cm^3^ of media supplemented with live yeast for 4 to 5 days. Males were reared separately to isotopically label all proteins^60^. In brief, embryos were collected and reared on an agar and sucrose media supplemented with heavy labeled (^13^C_6_^15^N_2_ arginine and ^13^C_6_^15^N_4_ lysine) yeast. Males were 8 to 14 days old and had mated at least once prior to this experiment. For mated samples females were mated *en masse* to an excess of heavy-labeled males. Mated females were kept on standard media for approximately 12 h to allow oviposition of accumulated, matured oocytes. Females were then transferred to media not conducive to oviposition (molasses and agar without yeast) for approximately 12 h to ensure the accumulation of oocytes that had matured in a postmating female.

Females were frozen at − 80^o^ C with a drop of water and stored until dissection. After thawing, ovaries were dissected away from all remaining tissues in approximately 20 females per sample in 1 × phosphate buffered saline (PBS). Ovarioles were then opened from the base to specifically isolate unfertilized, mature (stage 14) oocytes, which were identified by long and differentiated dorsal appendages^46^. Oocytes, including surrounding follicle cells of the egg chamber, were rinsed through a fresh drop of PBS and collected in a 1.5 ml Eppendorf tube with PBS. Approximately 100 oocytes were collected per sample, and three replicates were collected from both unmated and mated females. Samples were washed 3 × with PBS and solubilized in ~ 50 μL detergent (1 M HEPES with 2% SDS and 5% β-mercaptoethanol) with alternating cycles of heating (95 °C) and homogenization until completely solubilized. Prepared protein samples were stored at − 80 °C.

### Mass spectrometry

Protein isolation and tandem mass spectrometry (MS/MS) was conducted by Cambridge Proteomics following standard protocols, as previously described^61^. In brief, 30 μg of each sample was reduced (TCEP), alkylated (iodacetamide), trypsin digested, and labeled with 6-plex tandem mass tags (TMT, Thermo Scientific). Replicate oocyte samples from unmated females were labeled with 127N, 128N, 129C and those from mated females were labeled 129N, 130N, 130C. Resulting peptides were combined in equal volumes, cleaned and desalted on a Sep-Pak C18 Cartridge (Waters), and reconstituted in 0.1 mL 20 mM ammonium formate with 4% acetonitrile. Peptides were separated with high pH reverse-phase chromatography (Acquity UPLC bridged ethyl hybrid C18 column; 1.7 um particle, 2.1 mm; Waters) over 60 min (linear gradient 5–60% acetonitrile with 20 mM ammonium nitrate; flow rate 0.25 mL/min). Fractions were collected in 1 min increments dried and then resuspended in 0.1% formic acid and combined into 15 fractions.

Liquid chromatography with MS/MS was performed on a Dionex Ultimate 3000 rapid separation liquid chromatography nanoUPLC system (Thermo Scientific) coupled with a Lumos Orbitrap mass spectrometer. Peptides of each fraction were first loaded onto an Acclaim PepMap 100 C18 pre-column (5 µm particle size, 100 Å pore size, 300 µm inner diameter × 5 mm length, Thermo Scientific) with 0.1% formic acid for 3 min at 10ul/min. Eluted peptides were then separated on a reverse-phase nano EASY-spray column (PepMap C18; 2 µm particle size, 100 Å pore size, 75 µm inner diameter × 500 mm length, Thermo Scientific) for 90 min at 300nL/min in a gradient of 1.6% to 32% acetonitrile in 0.1% formic acid. Peptides from fractions were eluted from the column and sprayed (Easy-Spray Source; Thermo Fisher Scientific) into the mass spectrometer.

For each peptide ion, *m/z* values (MS1 scans) were measured at a resolution of 120,00 and range between 380 and 1500 Da. Data dependent MS/MS (MS2) scans (Top Speed) of the most abundant precursor ions (excluding those that were singly charged, had unassigned charge states, or were outside of the 70 s dynamic exclusion window) were isolated and fragmented by collision-induced disassociation (35% Normalized Collision Energy). From each MS2 scan the top 10 most abundant fragment ions were selected by Synchronous Precursor Selection for MS3 fragmentation by high energy collisional disassociation (65% normalized collision energy). For each fragment ion (mass range 100–500 Da) m/z values and relative abundances of reporter ions were measured in the Orbitrap analyzer (60,000 resolution).

### Protein identification and differential abundance analyses

Raw data files were processed using Proteome Discoverer v 2.3 (Thermo Fisher Scientific) and Mascot v 2.6 (Matrix Science), allowing for a MS tolerance of ± 10 ppm, MS/MS tolerance of ± 0.8 Da, and up to two missed tryptic cleavages. Peptides and proteins were identified in reference to a database of the longest isoform of the *D. melanogaster* genome (r6.21)^62^ accounting for common contaminant proteins (cRAP v 1.0; thegpm.org). Standard protein modifications of carbamidomethylation (cysteine, fixed), oxidation (methionine, variable) and deamidation (glutamine and arginine, variable) were included. The isotopic labelling of male proteins ensured that they were not identified in this analysis. Protein abundance estimates for each sample was calculated as the sum of centroid TMT receptor ions (± 2 millimass unit window) corrected for isotopic label purity. Labeling efficiency of TMT reporter ions was 99.8%.

In total, 761,774 MS/MS spectra were analyzed resulting in 87,683 peptide spectral matches and 5407 proteins. Heavy labelling of males prevented the detection of male-derived proteins and all proteomic differences can therefore be attributed to females. Proteins were filtered to include only *Drosophila* proteins that were high confidence in all samples (FDR ≤ 0.01) and identified by at least two unique peptides, for a total of 4,485 proteins. Protein intensities were log transformed and median difference normalized in MSnbase^63^. Differential abundance was calculated with empirical Bayes moderated t-tests using LIMMA^64^ and *p*-values were corrected for multiple comparisons using the Benjamini–Hochberg method. All analyses, following protein identification, were conducted in R.

### Statistical analysis, functional annotation, and data visualization

Pearson’s correlations between samples were visualized with complete-linkage hierarchical clustering heatmap in gplots. Sample relationships were also analyzed with a principal component analysis (PCA) using prcomp. Differential abundance plots were visualized with ggplot2. Departures from parity in the direction and magnitude of abundance changes were calculated with a weighted binomial test and Kruskal–Wallis test, respectively. The likelihood of observed overlaps between protein datasets was calculated using a cumulative weighted binomial distribution. Relationships between data sets were assessed using a Spearman’s correlation. Whether data sets had similar proportional changes was calculated with a chi-square test.

Functional enrichments were conducted with the Database for Annotation, Visualization and Integrated Discovery (DAVID) v 6.8^65^ with the *D. melanogaster* genome as the background and considered significant with an adjusted Benjamini–Hochberg of *p* < 0.05. Protein–protein interaction (PPI) networks and functional enrichment amongst differentially abundant proteins were analyzed and visualized using highest confidence (> 0.9 interaction score) interconnected proteins as implemented by the STRING database (v11) with all oocyte proteins designated as the background^66^.

## Supplementary Information

Supplementary Information 1.

Supplementary Information 2.

Supplementary Information 3.

Supplementary Information 4.

## Data Availability

Raw spectral files are available from the ProteomeXchange Consortium (PXD022142). Pre-computed protein intensities, differential abundance, and comparisons to existing data sets are available in Table S1. GO functional enrichments and network analyses are available in Table S2. Analysis code is available at github.com/CEMcDonoughGoldstein/OocyteProteome_FemaleMatingStatus.

## Abbreviations

FC: Fold change
GO: Gene ontology
PCA: Principal components analysis
PPI: Protein–protein interaction
