# Supplementary figures and images for: Drosophila oocyte proteome composition covaries with female mating status
Source: Sci Rep. 2021 Feb 4;11:3142. doi: 10.1038/s41598-021-82801-4 (PMC7862673; doi:10.1038/s41598-021-82801-4)

Fig S1

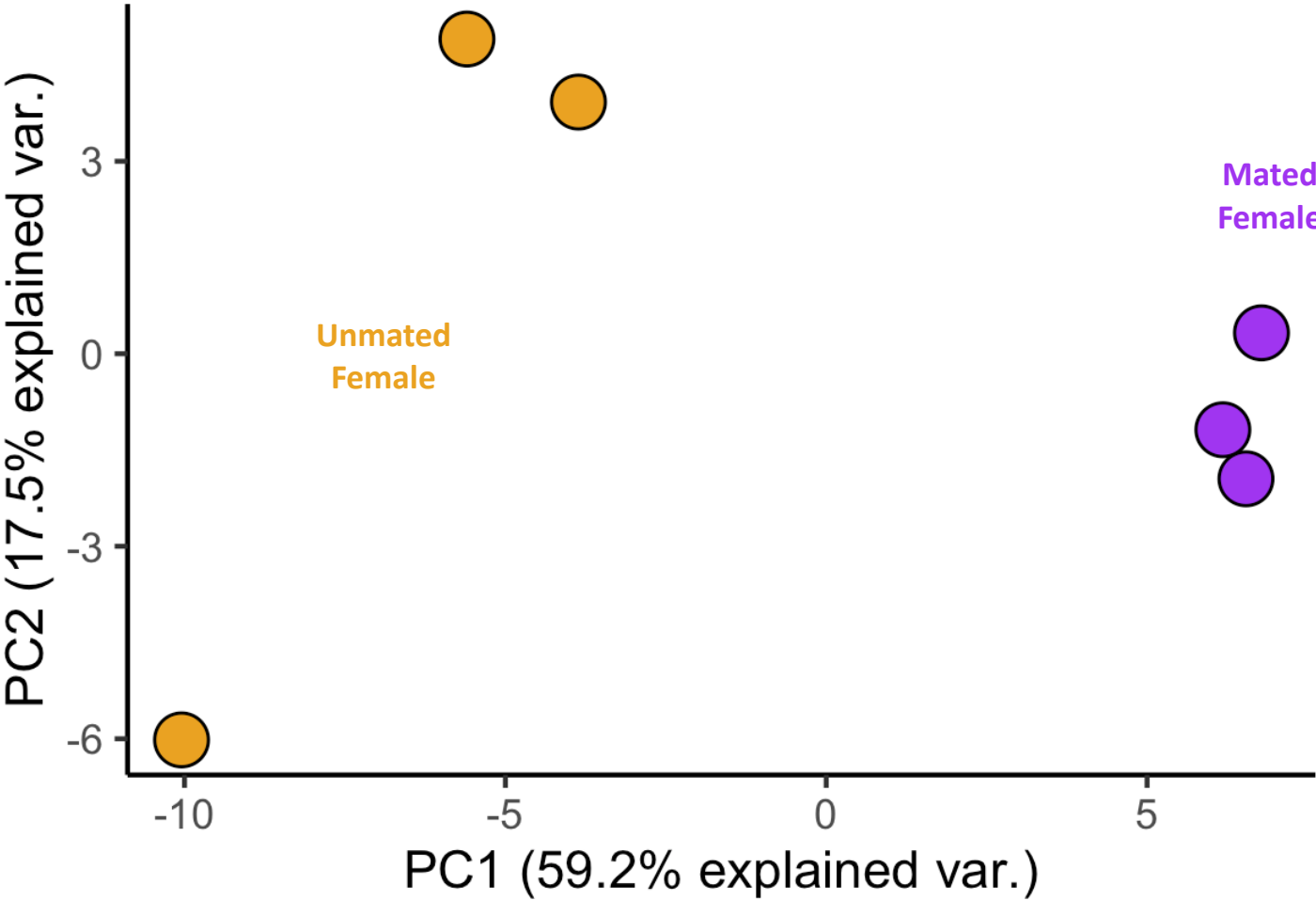

Fig S2

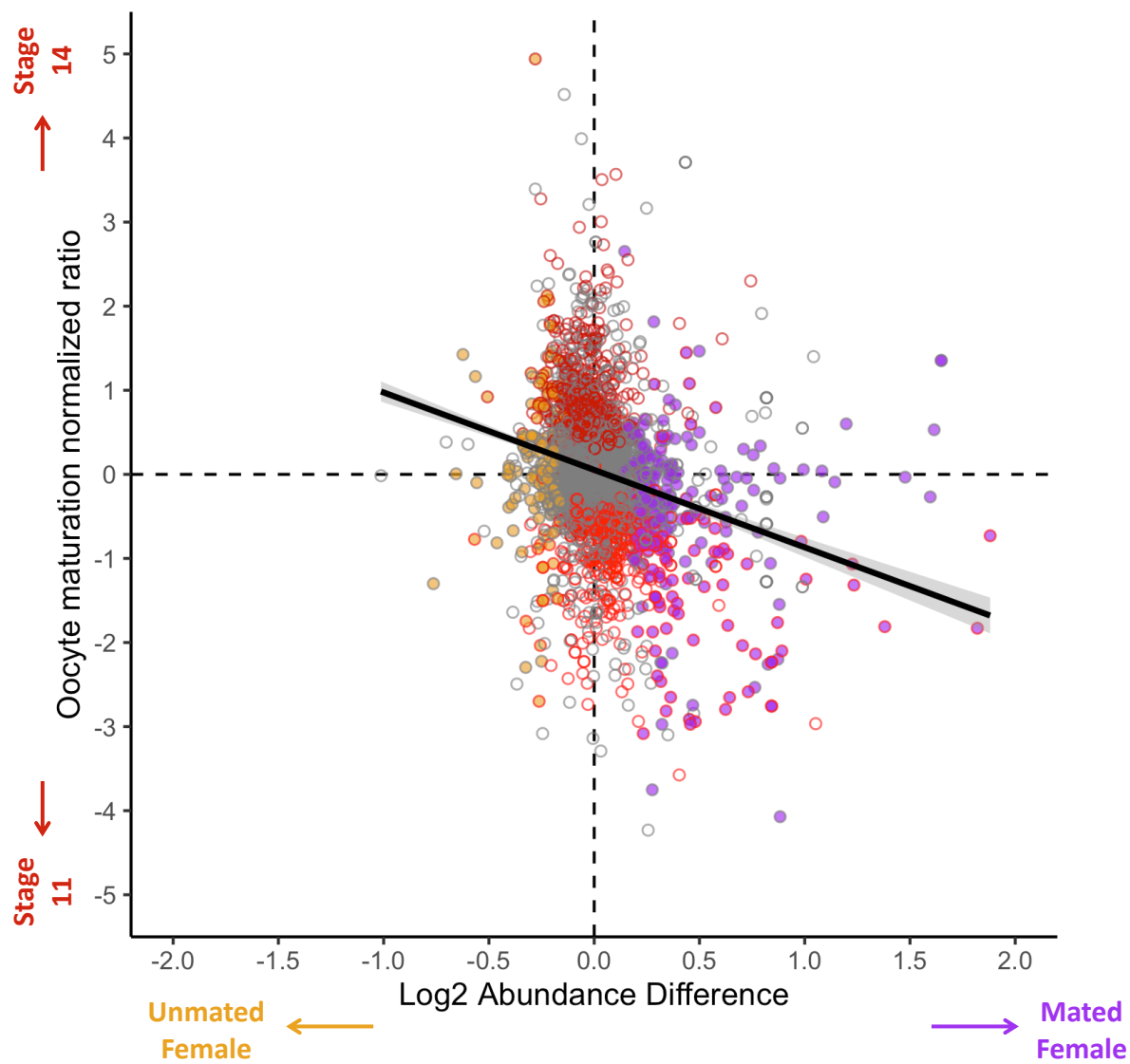

Supplement: Supplementary file 1 — Supplementary Information 1. [file 41598_2021_82801_MOESM1_ESM.pdf]
