# Supplementary material for: Drosophila oocyte proteome composition covaries with female mating status
Source: Sci Rep. 2021 Feb 4;11:3142. doi: 10.1038/s41598-021-82801-4 (PMC7862673; doi:10.1038/s41598-021-82801-4)
Supplement: Supplementary file 2 — Supplementary Information 2. [file 41598_2021_82801_MOESM2_ESM.docx]

**Female mating status influences protein composition of mature *Drosophila* oocytes**

Caitlin E. McDonough-Goldstein^1^*, Scott Pitnick^1^ and Steve Dorus^1^*

^1^ Center for Reproductive Evolution, Biology Department, Syracuse University, Syracuse, NY, USA

*Corresponding authors: Caitlin E. McDonough-Goldstein (mcdonouce@gmail.com); Steve Dorus (sdorus@syr.edu)

**- Supplementary Information**

**Supplemental Fig 1** Principal component analysis. The first Principal Component (PC) explains the majority (59.2%) of variation among samples and separates replicates of oocytes from unmated females (negative PC1 loading values) from oocytes from mated females (positive PC1 loading values).

**Supplemental Fig 2** Correlation between log2 difference in protein abundance in oocytes from mated and unmated females and average normalized protein abundance during oocyte maturation (stage 11 versus stage 14) from ^33^. Proteins in red were differentially abundant during maturation, proteins in purple had greater abundance in oocytes from mated females and yellow from unmated females. There was a significant, but weak, negative correlation (black line with confidence interval in grey, adj. R^2^ = 0.058, *p* < 0.001). The greatest overlap in protein changes are those with significant decrease during maturation and greater abundance in oocytes from mated females.

**Table S1** Protein abundance and summary of analysis

**Table S2** Functional enrichments for differentially abundant proteins
